# Applicability of DRIS in bananas based on the accuracy of nutritional diagnoses for nitrogen and potassium

**DOI:** 10.1038/s41598-022-22554-w

**Published:** 2022-10-27

**Authors:** Diego Villaseñor-Ortiz, Renato de Mello Prado, Gilmara Pereira da Silva, Luis Felipe Lata-Tenesaca

**Affiliations:** 1 Department of Agricultural Production Sciences, São Paulo State University “Júlio de Mesquita Filho” (UNESP), Jaboticabal, SP 14884-900 Brazil; 2 School of Agricultural Sciences, Technical University of Machala (UTMACH), 070223 Machala, El Oro Ecuador; 3 Department of Agronomy, State University of Roraima - UERR, University Campus of Rorainópolis, Rorainópolis, 69317-805 Brazil; 4 Department of Plant Pathology, Federal University of Viçosa (UFV), Viçosa, MG 36570-090 Brazil

**Keywords:** Plant development, Plant stress responses

## Abstract

DRIS (Diagnosis Recommendation Integrated System) is a tool used in the interpretation of leaf analyses that values the balance of nutrients, an important fact for a better assessment of the nutritional status of banana plants. Its usefulness depends on the ability to identify the nutrients that limit productivity in order to correct possible nutritional imbalances, but there is a lack of research in all crops, including bananas, to assess the accuracy of these diagnoses, which have a worrying global implication. To this end, this study evaluates DRIS norms for banana cultivation in Ecuador and the use of accuracy measurements for nutritional diagnosis, verifying the capacity of DRIS to detect true nutritional status based on plant response. The database created here contains 233 results referring to productivity and leaf contents of N, P, K, Ca, Mg, S, Cl, Fe, Mn, Cu, B, and Zn for banana trees in 2018, 2019, and 2020. Then, a field experiment evaluated doses of nitrogen and potassium and the accuracy of DRIS norms for N and K. The results show that the DRIS of banana produced in Ecuador depends on the nutrient being variable according to the crop nutritional status. The DRIS norms for diagnosis of N and K result in an acceptable accuracy to identify only deficiencies and toxicities, respectively, indicating the need for adjustments in these standards for later use in the field. Thus, there is a need for more research aiming to adopt calibrated DRIS diagnostic norms to assess the nutritional status of bananas in Ecuador.

## Introduction

Banana (*Mussa* spp.) is a fast-growing plant with a high nutritional demand, mainly for potassium (K) and nitrogen (N)^1^. Such high nutritional demand is important to ensure the biological functions of these nutrients in the plant, therefore, it is a responsive species to the application of these nutrients^2^.

In these circumstances, the assessment of the nutritional status for bananas, shows states of sufficiency, deficiency, or excess of nutrients that directly interfere with fruit productivity^3^. For this, the use of diagnostic methods that compare the results of leaf levels of nutrients in a sample with the appropriate nutritional standards obtained in crops with a high productivity is essential^4^, and may help in designing efficient nutrient management practices for improved crop yield^5^.

This procedure for interpreting leaf nutritional values for bananas has traditionally been based on the standards critical level (CL) and/or sufficiency range (SR)^6^, where, according with these authors, each nutrient is considered alone (univariate relationship) and not considering possible interactions between them^7^.

However, an isolated consideration of nutrients may lead to interpretation issues^8^, since values equal to or less than CL or SR are not always associated with a high or low productive yield, respectively. That errors in diagnoses may occur due to the use of outdated leaf nutrient standards or data obtained in other locations than the cultivation area that is the target of the diagnosis^9^. Given the limitations of conventional methods, alternative nutrient diagnostic approaches are needed to rationalize fertilizer investment decisions^10^.

An alternative to minimize problems of CL or sufficiency range is the method of the Diagnosis and Recommendation Integrated System (DRIS). The DRIS method uses bivariate relationships between nutrients, generating DRIS norms with a high applicability since it can be used in the cultivation region intended for leaf diagnosis with a low implementation cost in relation to the CL system^7^.

For example, some authors used the DRIS method, to obtain nutritional diagnostic standards for banana cultivation in some regions of the world^11–15^. The database of leaf samples and productivity ranged from 68 to 915 observations.

Regardless of the method used to interpret the results of leaf analysis, its usefulness depends on the ability to make true predictions of nutritional diagnosis. In this context, nutritional diagnostic accuracy measurements are designed to meet the need to verify the accuracy of diagnostic standards developed using the DRIS method.

For this, Beverly and Hallmark^16^ propose the first methodology, called "Prescient Diagnostic Analysis Methodology", which comprises three measurements of accuracy and an accuracy ratio. This method has limitations because, for a high accuracy of a certain nutrient, it is enough to have a high percentage of true diagnoses for balance even if there is no true diagnosis for insufficiency.

To improve the accuracy test, Wadt and Lemos^17^ proposed a method that expanded to seven measurements of accuracy considering three nutritional states (deficiency, sufficiency, and excess) and which, according to Wadt^18^, adapts itself to other methodologies compatible with the use of the criterion of potential response to fertilization (PRF).

In this context, there is one research that uses diagnostic methods that perform a precision test; one of them is that of Teixeira et al.^19^, who used the accuracy method proposed by Beverly and Hallmark^16^ to evaluate the performance of CL and DRIS for N and K. The authors found that the accuracy in relation to N, the efficiency of the diagnoses based on CL was 48%; in relation to DRIS, it was 69%. For K, the efficiency of diagnoses using DRIS and CL was 63%.

Regardless of the country, there is a need for further research to assess the performance of DRIS norms in different regions of the world aiming the diagnosis of N and K, which may be low or acceptable for one element or for two nutrients, and to propose changes if there is a need to improve the quality of diagnoses that affect the increase in the adoption of leaf analysis in crops (however, there is a lack of research on this topic).

In this context, considering the nutritional diagnoses of N and K, we hypothesize that the adoption of the accuracy measurements proposed by Wadt and Lemos^17^ is efficient to measure the accuracy level of DRIS norms and that there may still be a variation in the number of correct values when the diagnosis indicates deficiency, sufficiency, or excess depending on the nutrient. For this purpose, we aim to establish DRIS norms for Ecuador and to evaluate the accuracy of nutritional diagnoses of N and K, based on the culture's response to the fertilization of these nutrients. Our proposal constitutes a new approach to evaluate the DRIS tested in banana but the systematic has a global reach as it can be applied for an accurate analysis of the DRIS of other crops.

## Results

The database containing results of leaf analysis and annual productivity was subdivided into populations of high and low productivity (HP and LP). The mean productivity (PR) of the HP subpopulation was significantly (P ≤ 0.05) higher than that of the LP subpopulation (Table 1). The concentrations of N, P, K, and Cl were on average 2.41, 0.18, 3.47, 1.12% and the concentrations of Mn, Cu, Zn, and B were on average 218, 10.1, 21.4 and 20.2 mg kg^− 1^, respectively, in the HP subpopulation; the Mg of 0.3% and the Fe of 86.3% resulted in a higher concentration in the LP subpopulation. In this context, the means of PR (60.47 t ha^− 1^), K (3.5%) and Cl (1.56%) presented the highest values for HP, while S (0.17%) and Mn (484.8 mg kg^− 1^) did not differ significantly between the two subpopulations.

**Table 1:** Means, standard deviation and Shapiro–Wilk normality test of productivity and concentration of nutrients for low and high productivity subpopulations obtained in the database aiming to determine the initial and field experiment data for DRIS norms.

	Subpopulation of low productivity (LP)				Subpopulation of high productivity (HP)			
	x̄	*σ*	W	*p*	x̄	*σ*	W	*p*
**IBD**								
Productivity, t ha^− 1^	37.40	10.06	0.9591	0.000027	60.08*	3.32	0.9555	0.08822^+^
N, %	2.25	0.27	0.9874	0.093511^+^	2.41*	0.24	0.9597	0.14394^+^
P, %	0.17	0.02	0.9693	0.000484	0.18*	0.02	0.9660	0.21664^+^
K, %	3.08	0.49	0.9895	0.187565^+^	3.47*	0.42	0.9694	0.31563^+^
Mg, %	0.30*	0.04	0.9917	0.378431^+^	0.28	0.05	0.9073	0.00210
Ca, %	0.73	0.17	0.9892	0.172239^+^	0.74^ ns^	0.20	0.9757	0.47144^+^
S, %	0.15	0.03	0.9671	0.000708	0.16^ ns^	0.03	0.9640	0.39115^+^
Cl, %	1.03	0.20	0.9765	0.008993	1.12*	0.23	0.8377	0.00263
Fe, mg kg^− 1^	86.3*	20.4	0.9674	0.000485	80.8	15.9	0.8823	0.00116
Mn, mg kg^− 1^	189.4	67.0	0.9744	0.001826	218.0*	73.8	0.9604	0.16185^+^
Cu, mg kg^− 1^	8.6	2.7	0.9624	0.000108	10.1*	2.8	0.9496	0.12932^+^
Zn, mg kg^− 1^	18.8	3.7	0.9403	0.000001	21.4*	3.9	0.9777	0.58798^+^
B, mg kg^− 1^	18.2	8.5	0.9536	0.000035	20.2*	5.3	0.9872	0.91747^+^
**EBD**								
Productivity, t ha^− 1^	47.42	5.33	0.9591	0.06406^+^	60.47*	2.20	0.9580	0.74633^+^
N, %	2.93	0.52	0.9874	0.00004	2.84^ ns^	0.57	0.9180	0.30193^+^
P, %	0.18	0.02	0.9693	0.00414	0.18^ ns^	0.02	0.9579	0.74520^+^
K, %	2.99	0.48	0.9895	0.03598	3.50*	0.24	0.8744	0.08819^+^
Mg, %	0.32	0.04	0.9917	0.82025^+^	0.31^ ns^	0.04	0.9487	0.62799^+^
Ca, %	0.76	0.15	0.9892	0.00508	0.73^ ns^	0.13	0.9420	0.54376^+^
S, %	0.17*	0.01	0.9671	0.00108	0.16	0.02	0.9807	0.96973^+^
Cl, %	1.44	0.20	0.9765	0.21016^+^	1.56*	0.11	0.9406	0.52704^+^
Fe, mg kg^− 1^	90.6	13.6	0.9674	0.03140	88.8^ ns^	12.1	0.9910	0.99841^+^
Mn, mg kg^− 1^	484.8*	298.4	0.9744	0.00000	339.9	175.1	0.7390	0.00262
Cu, mg kg^− 1^	8.2	1.8	0.9624	0.00104	7.8^ ns^	1.7	0.8950	0.16026^+^
Zn, mg kg^− 1^	21.7	1.2	0.9403	0.91959^+^	21.2^ ns^	1.3	0.9126	0.29909^+^
B, mg kg^− 1^	22.0	1.4	0.9536	0.92948^+^	21.3^ ns^	1.1	0.9216	0.40524^+^

^+^denotes a normal population distribution.

*means between the LP and HP subpopulations are significantly different at ≤ 0.05 using the F test for variances of two samples.

*IBD* initial data base for DRIS norms, *EBD* experiment data base for DRIS norms from.

The dual relationships of nutritional contents (Table 2) obtained for the HP population, transformed into a logarithmic scale and the respective standard deviations. The dual relationships are diagnostic references for the region under study. Using these norms, we calculated DRIS indexes (I_DRIS) for each nutrient.

**Table 2:** Mean (x̄) and standard deviation (σ) of the resulting bivariate nutritional association of leaf dry matter samples obtained from the high productivity subpopulation of banana (*Mussa* spp.) for the establishment of DRIS standards.

	N	P	K	Mg	Ca	S	Cl	Fe	Mn	Cu	Zn	B
N/	**–**											
x̄	**–**	1.12	− 0.16	0.93	0.52	1.16	0.32	1.48	1.08	2.39	2.05	2.10
*σ*	**–**	0.24	0.08	0.25	0.17	0.57	0.17	0.61	0.38	1.11	0.67	0.68
P/		**–**										
x̄	− 1.12	**–**	− 1.29	− 0.19	− 0.59	0.05	− 0.80	0.35	− 0.06	1.28	0.93	0.97
*σ*	0.24	**–**	0.28	0.08	0.15	0.09	0.41	0.17	0.17	0.58	0.26	0.27
K/			**–**									
x̄	0.16	1.29	**–**	1.09	0.68	1.33	0.50	1.63	1.22	2.55	2.21	2.26
*σ*	0.08	0.28	**–**	0.25	0.20	0.65	0.26	0.67	0.39	1.16	0.72	0.74
Mg/				**–**								
x̄	− 0.93	0.19	− 1.09	**–**	− 0.40	0.25	− 0.63	0.54	0.13	1.47	1.12	1.16
*σ*	0.25	0.08	0.25	**–**	0.18	0.16	0.32	0.23	0.17	0.67	0.35	0.36
Ca					**–**							
x̄	− 0.52	0.59	− 0.68	0.40	**–**	0.62	− 0.17	0.96	0.54	1.85	1.53	1.57
*σ*	0.17	0.15	0.20	0.18	**–**	0.31	0.13	0.41	0.23	0.82	0.41	0.45
S/						**–**						
x̄	− 1.16	− 0.05	− 1.33	− 0.25	− 0.62	**–**	− 0.62	− 0.85	0.28	− 0.12	1.24	0.89
*σ*	0.57	0.09	0.65	0.16	0.31	**–**	0.31	0.44	0.17	0.13	0.63	0.44
Cl/							**–**					
x̄	− 0.32	0.80	− 0.50	0.63	0.17	0.85	**–**	1.10	0.78	2.05	1.72	1.75
*σ*	0.17	0.41	0.26	0.32	0.13	0.44	**–**	0.56	0.41	0.99	0.87	0.89
Fe/								**–**				
x̄	− 1.48	− 0.35	− 1.63	− 0.54	− 0.96	− 0.28	− 1.10	**–**	− 0.40	0.89	0.57	0.62
*σ*	0.61	0.17	0.67	0.23	0.41	0.17	0.56	**–**	0.24	0.46	0.26	0.27
Mn/									**–**			
x̄	− 1.08	0.06	− 1.22	− 0.13	− 0.54	0.12	− 0.78	0.40	**–**	1.37	0.99	1.02
*σ*	0.38	0.17	0.39	0.17	0.23	0.13	0.41	0.24	**–**	0.65	0.39	0.41
Cu/										**–**		
x̄	− 2.39	− 1.28	− 2.55	− 1.47	− 1.85	− 1.24	− 2.05	− 0.89	− 1.37	**–**	− 0.33	− 0.30
*σ*	1.11	0.58	1.16	0.67	0.82	0.63	0.99	0.46	0.65	**–**	0.19	0.23
Zn/											**–**	
x̄	− 2.05	− 0.93	− 2.21	− 1.12	− 1.53	− 0.89	− 1.72	− 0.57	− 0.99	0.33	**–**	0.03
*σ*	0.67	0.26	0.72	0.35	0.41	0.44	0.87	0.26	0.39	0.19	**–**	0.13
B/												**–**
x̄	− 2.10	− 0.97	− 2.26	− 1.16	− 1.57	− 0.88	− 1.75	− 0.62	− 1.02	0.30	− 0.03	**–**
*σ*	0.68	0.27	0.74	0.36	0.45	0.45	0.89	0.27	0.41	0.23	0.13	**–**

We used the criterion of evaluating the nutritional diagnosis of a treatment with the application of nutrients to compare 48 cases of fertilization with N and K_2_O (Tables 3 and 4). We took the first case of each element as an example to explain the procedure: the diagnosis of the control treatment (0 kg ha^− 1^ of N) obtained through the I_DRIS (Table 2) resulted in a balanced nutritional state according to the crop potential response to fertilization (PRF) calculation process: 43.6 t ha^− 1^ of production. We compared this treatment with the productivity reflected in the application of 200 kg ha^− 1^ of N, with a level of 45 t ha^− 1^, which represented an increase of 3% in production. This increase is due to fertilization with N and indicates that the plant indeed was in a nutritional equilibrium (Eq) since it did not exceed the 10% proposed in the methodology. This allows us to infer that the Eq diagnosis is true, and the calculated productivity gain was 1.4 t ha^− 1^ (Table 3). Likewise, we performed the calculation for the K element: the diagnosis of the control treatment (0 kg ha^− 1^ of K_2_O) obtained through the I_DRIS (Table 2) resulted in a nutritional deficiency (D), with 43.6 t ha^− 1^ of production, compared to the treatment with application of K (375 kg ha^− 1^). The increase in productivity is 51.6 t ha^− 1^ (increase of 18%), which indicates a true diagnosis of deficiency since the application of fertilization directly affected the increase in production (Table 4).

**Table 3:** Accuracy of nutritional diagnosis for the nutrient N for DRIS norms in banana (*Mussa* spp.).

Case	Comparison	Prod. control	Prod. manured	Gain in Prod	Increase in Prod	Diagnosis Control	Diagnosis Productivity
	(N kg ha^− 1^)	(t ha^− 1^)	(t ha^− 1^)	(t ha^− 1^)	(%)	DRIS	PRF
1	0 × 200	43.6	45.0	1.4	3	Eq	Eq
2	0 × 200	40.9	44.9	4.0	10	Eq	Eq
3	0 × 200	40.6	42.3	1.7	4	Eq	Eq
4	0 × 200	40.4	40.1	− 0.3	− 1	Eq	Eq
5	0 × 200	51.6	51.9	0.3	1	Eq	D
6	0 × 200	50.5	51.0	0.5	1	Eq	Eq
7	0 × 200	49.7	50.5	0.8	2	Eq	Eq
8	0 × 200	49.5	50.0	0.5	1	Eq	Eq
9	0 × 200	62.8	64.2	1.3	2	Eq	D
10	0 × 200	61.2	62.2	1.0	2	Eq	D
11	0 × 200	58.6	59.6	1.1	2	Eq	Eq
12	0 × 200	57.2	56.0	− 1.3	− 2	Eq	Eq
13	0 × 200	44.1	49.7	5.6	13	D	Eq
14	0 × 200	39.0	49.6	10.6	27	D	D
15	0 × 200	38.2	49.6	11.3	30	D	D
16	0 × 200	38.0	46.9	8.9	23	D	D
17	200 × 400	45.0	46.8	1.8	4	Eq	Ex
18	200 × 400	44.9	43.9	− 1.1	− 2	Eq	Ex
19	200 × 400	42.3	43.6	1.3	3	Eq	Ex
20	200 × 400	40.1	42.5	2.4	6	Eq	Ex
21	200 × 400	51.9	55.3	3.5	7	Eq	Eq
22	200 × 400	51.0	53.1	2.1	4	Eq	Eq
23	200 × 400	50.5	52.0	1.5	3	Eq	Eq
24	200 × 400	50.0	48.9	− 1.1	− 2	Eq	Eq
25	200 × 400	64.2	58.6	− 5.6	− 9	Eq	Eq
26	200 × 400	62.2	56.0	− 6.3	− 10	Ex	Eq
27	200 × 400	59.6	55.9	− 3.7	− 6	Eq	Ex
28	200 × 400	56.0	54.6	− 1.3	− 2	Eq	Eq
29	200 × 400	49.7	49.9	0.2	0	Eq	Eq
30	200 × 400	49.6	48.3	− 1.3	− 3	Eq	Ex
31	200 × 400	49.6	47.9	− 1.6	− 3	Eq	Eq
32	200 × 400	46.9	45.2	− 1.7	− 4	Eq	Eq
33	400 × 600	46.8	46.2	− 0.6	− 1	Eq	Ex
34	400 × 600	43.9	45.1	1.3	3	Eq	Ex
35	400 × 600	43.6	44.7	1.1	2	Eq	Ex
36	400 × 600	42.5	43.1	0.6	1	Eq	Ex
37	400 × 600	55.3	56.0	0.7	1	Eq	Eq
38	400 × 600	53.1	54.8	1.7	3	Eq	Ex
39	400 × 600	52.0	53.5	1.5	3	Eq	Ex
40	400 × 600	48.9	49.5	0.5	1	Eq	Eq
41	400 × 600	58.6	61.4	2.9	5	Eq	Eq
42	400 × 600	56.0	61.1	5.1	9	Eq	Eq
43	400 × 600	55.9	58.2	2.3	4	Eq	Eq
44	400 × 600	54.6	55.7	1.0	2	Eq	Ex
45	400 × 600	49.9	44.8	− 5.1	− 10	Ex	Ex
46	400 × 600	48.3	42.9	− 5.4	− 11	Ex	Eq
47	400 × 600	47.9	41.3	− 6.6	− 14	Ex	Eq
48	400 × 600	45.2	37.9	− 7.3	− 16	Ex	Eq

*N* nitrogen dose, *Prod* average productivity, *Eq* nutritional equilibrium, *D* nutritional deficiency, *Ex* nutritional excess, *PRF* potential response to fertilization.

**Table 4:** Accuracy of nutritional diagnosis for the nutrient K for DRIS norms in banana (*Mussa* spp.).

Case	Comparison	Prod. Control	Prod. manured	Gain in Prod	Increase in Prod	Diagnosis Control	Diagnosis Productivity
	(K kg ha^− 1^)	(t ha^− 1^)	(t ha^− 1^)	(t ha^− 1^)	(%)	DRIS	PRF
1	0 × 375	43.6	51.6	8.0	18	D	D
2	0 × 375	40.9	50.5	9.6	24	D	D
3	0 × 375	40.6	49.7	9.1	22	D	D
4	0 × 375	40.4	49.5	9.1	23	D	D
5	0 × 375	45.0	51.9	6.9	15	D	Eq
6	0 × 375	44.9	51.0	6.1	14	D	D
7	0 × 375	42.3	50.5	8.2	19	D	D
8	0 × 375	40.1	50.0	9.9	25	D	Eq
9	0 × 375	46.8	55.3	8.6	18	D	D
10	0 × 375	43.9	53.1	9.3	21	D	D
11	0 × 375	43.6	52.0	8.4	19	D	Ex
12	0 × 375	42.5	48.9	6.4	15	D	Ex
13	0 × 375	46.2	56.0	9.9	21	D	D
14	0 × 375	45.1	54.8	9.7	22	D	D
15	0 × 375	44.7	53.5	8.8	20	D	D
16	0 × 375	43.1	49.5	6.3	15	D	D
17	375 × 750	51.6	62.8	11.2	22	D	Eq
18	375 × 750	50.5	61.2	10.7	21	D	Eq
19	375 × 750	49.7	58.6	8.8	18	D	Eq
20	375 × 750	49.5	57.2	7.8	16	D	Eq
21	375 × 750	51.9	64.2	12.3	24	D	D
22	375 × 750	51.0	62.2	11.3	22	D	D
23	375 × 750	50.5	59.6	9.1	18	D	Ex
24	375 × 750	50.0	56.0	6.0	12	D	D
25	375 × 750	55.3	58.6	3.2	6	Eq	Eq
26	375 × 750	53.1	56.0	2.9	5	Eq	D
27	375 × 750	52.0	55.9	3.9	7	Eq	D
28	375 × 750	48.9	54.6	5.7	12	D	D
29	375 × 750	56.0	61.4	5.4	10	Eq	D
30	375 × 750	54.8	61.1	6.3	11	D	Ex
31	375 × 750	53.5	58.2	4.7	9	Eq	D
32	750 × 1125	49.5	55.7	6.2	13	D	D
33	750 × 1125	62.8	46.2	− 16.7	− 27	Ex	Eq
34	750 × 1125	61.2	45.1	− 16.1	− 26	Ex	Eq
35	750 × 1125	58.6	44.7	− 13.9	− 24	Ex	Eq
36	750 × 1125	57.2	43.1	− 14.1	− 25	Ex	Eq
37	750 × 1125	64.2	56.0	− 8.1	− 13	Ex	D
38	750 × 1125	62.2	54.8	− 7.4	− 12	Ex	Ex
39	750 × 1125	59.6	53.5	− 6.1	− 10	Ex	D
40	750 × 1125	56.0	49.5	− 6.5	− 12	Ex	Ex
41	750 × 1125	58.6	61.4	2.9	5	Eq	D
42	750 × 1125	56.0	61.1	5.1	9	Eq	Ex
43	750 × 1125	55.9	58.2	2.3	4	Eq	D
44	750 × 1125	54.6	55.7	1.0	2	Eq	D
45	750 × 1125	61.4	44.8	− 16.6	− 27	Ex	D
46	750 × 1125	61.1	42.9	− 18.2	− 30	Ex	D
47	750 × 1125	58.2	41.3	− 16.8	− 29	Ex	Ex
48	750 × 1125	55.7	37.9	− 7.8	− 32	Ex	D

*K* potassium dose, *Prod* average productivity, *Eq* nutritional equilibrium, *D* nutritional deficiency, *Ex* nutritional excess, *PRF* potential response to fertilization.

Based on the analysis of accuracy measurements, which indicates the distribution of the diagnostic cases of nutritional status (Table 5), we observed, before the calculation of the accuracy measurement of nutritional diagnoses, that the global accuracy (GA) results in a value of 51 and 33% for N and K, respectively (Table 6). The accuracy measurement of true diagnostics for insufficiency (ACI)showed a performance of 89 and 48% of the diagnoses evaluated for N and K, respectively. For the accuracy measurement of true diagnoses for equilibrium (ACEq), the accuracy of the diagnoses was slightly greater than 50%, reaching 51% for N and 52% for K. The accuracy measurement of true diagnoses for excess (ACEx) resulted in a good performance, with a correct diagnosis for N and K of 62 and 66%, respectively.

**Table 5:** Counting of the nutritional diagnosis for potassium and nitrogen nutrients determined by DRIS standards in bananas (*Mussa* spp).

Nutrient	Diagnosis											
N	IT	EqT	ExT	F_D_	Eq	FEx	IF_(S)_	IF_(T)_	EqF_(D)_	Eq F_(T)_	ExF_(D)_	ExF_(S)_
Count	3	23	1	1	16	4	3	0	1	4	0	13
%	4	33	1	1	23	6	4	0	1	6	0	19
K												
Count	17	1	3	10	8	9	7	5	6	4	4	1
%	23	1	4	13	11	12	9	7	8	5	5	1

*IT* count of the number of true diagnoses for nutritional insufficiency, *EqT *count of the number of true diagnoses for nutritional balance, *ExT* count of the number of true diagnoses for nutritional excess, *IF*_*(S)*_ count of the number of false diagnoses for insufficiency in situations of recognized nutritional sufficiency, *IF*_*(T)*_ count of the number of false diagnoses for insufficiency in situations of recognized nutritional toxicity, *EqF*_*(D)*_ counting the number of false diagnoses for balance in situations of recognized nutritional deficiency, *EqF*_*(T)*_ count of the number of false diagnoses for balance in situations of recognized nutritional toxicity, *ExF*_*(D)*_ count of the number of false diagnoses for excess in situations of recognized nutritional deficiency, *ExF*_*(S)*_ count of the number of false diagnoses for excess in situations of recognized nutritional sufficiency, *F*_*(D)*_ count of the number of false diagnoses for excess in plants with nutritional deficiency, *Eq* total diagnoses for nutritional balance, *FEx* total diagnoses for nutritional excess.

**Table 6:** Accuracy of nutritional diagnosis for the nutrient potassium and nitrogen for DRIS norms in banana (*Mussa* spp).

Nutrient	Diagnosis							
	GA	ACI	ACEQ	ACEX	ACD	ACS	ACT	t ha^− 1^
N	0.51	0.89	0.51	0.62	0.91	0.25	0.70	0.63
K	0.33	0.48	0.52	0.66	0.54	0.59	0.54	2.14

*GA* global accuracy, *ACI* accuracy measurement of true diagnoses for insufficiency, *ACEQ* accuracy measurement of true diagnoses for equilibrium, *ACEX* accuracy measurement of true diagnoses for excess, *ACD* accuracy measurement of true diagnoses for deficiency, *ACS* accuracy measurement of true diagnoses for sufficiency, *ACT* accuracy measurement of true diagnoses for toxicity.

As for accuracy measurement of true diagnoses for deficiency (ACD), the diagnostic correctness was high, reaching 91% for N and 54% for K. However, the DRIS method used in the precise diagnoses for accuracy diagnoses for sufficiency (ACS) had a low performance, especially for N (25%) in relation to the K, which had 59% of correctness. The DRIS method showed a good efficiency for predicting accuracy diagnoses for toxicity (ACT), especially for N (70%), compared to K (54%), which had a negligible efficiency.

## Discussion

Data partition at the highest inflection point (54.5 and 56.7 t ha^− 1^) in both populations of data analyzed placed 19 and 17% of the observations in the high-productivity population, respectively (Tables 4 and 5). The values suggest that the approach of the total population average + 1 × standard deviation is applicable to this study, as Silva et al.^20^ proposed. However, it was not applicable for the division of populations in other studies where the inflection points were beyond the range of observations^13^. Likewise, Abebe et al.^21^ argue that the differentiation between high- and low-productivity populations are good indicators for DRIS diagnostic standards.

The estimates Beaufils^7^ proposed are based on the fact that the larger the size of a population sample, the better the appreciation of the mean and the variability of the sample of interest (Tables 4 and 5). For this reason, Maroccos et al.^22^ suggests using large databases to obtain more accurate information, with consistent diagnoses with the reality of the studied system. However, Mourão Filho^23^ consider that the quality of a set of DRIS norms is not restricted only to the database, but also to the quality of the data records obtained from the levels of leaf nutrients and productivity.

The fact that the HP subpopulation had a significantly larger content of N, P, K, Zn, and B (P ≤ 0.05) in the initial database (Table 4) agrees with the observations of Teixeira et al.^12^ and Wairegi and van Asten^13^ regarding data from *Mussa* spp., analyzed in Brazil and Uganda, respectively. The authors concluded that low productivity levels are more often associated with low nutritional contents.

In this sense, high levels of nutrients in the plant tissue are related to the plant's requirement, that is, the amount required to meet biological functions^9^, which manifest as increases in productivity. In addition, Wairegi and van Asten^13^ state that, since there are differences between nutritional levels in both subpopulations, there is an adequate reliability in the generation of DRIS norms. The high nutritional levels in the LP population of the experiment (Table 5) can be explained by the concentration effect due to a reduced relative growth rate of dry matter, which accumulates nutrients in the plant tissue. This fact stresses the fragility of a diagnosis using univariate methods, indicating sufficiency when in fact there is a state of deficiency**.** Franco-Hermida et al.^24^ described similar reports in the culture of cut roses (*Rosa* spp. L.).

The nutritional means observed (Tables 4 and 5), together with the established standards (Table 6), are not similar to those observed in other studies on banana cultivation, such as Wortmann et al.^11^ in Tanzania with an unmentioned cultivar, Teixeira et al.^12^ in Brazil, and Angeles et al.^8^ in the Philippines; both studies used the cultivar Gros Michel (AAA). These differences in nutrient content affect DRIS norms because, in each study, the edaphoclimatic conditions are different. Rodrigues Filho et al.^15^ studied in two different Brazilian regions and added that differences in soil fertility, as well as in non-nutritional factors, interfere with DRIS norms. In light of this, the importance of specific DRIS norms to diagnose nutrient imbalances in banana plantations in southern Ecuador is evident. However, Wairegi and van Asten^13^ add that the DRIS norms applied to banana trees are not specific for the genomic group AAA. Normally, DRIS norms for a given crop are not created for a single one cultivar but for the species, with the expectation that this may not affect the diagnosis. However, research on this topic is scarce.

When evaluating the diagnostic accuracy measurements for the nutrients N and K, there were low GA values (Table 6), a fact also highlighted by Wadt and Lemos^17^. Regarding the levels found in the correction degrees for ACI, we observed an adequate level of 89% for N, quite above that of K (48%) (Table 6). According to Wadt and Lemos^17^, a fertilization recommendation above the real requirements shows a state of insufficiency in nutritionally-balanced plants.

The ACEQ measurement, in this study, reflects that about 50% of the diagnoses of N and K were correct as for nutritional balance, a fact Silva et al.^25^ also reported. Wadt and Lemos^17^ adds that true diagnoses for nutritional balance are important to avoid environmental impacts due to excessive use of fertilizers.

Regarding the ACEx accuracy measurement, the values above 60% for N and K indicate the degree of correctness of the diagnosis of nutritional excess (Table 6). This result is possibly due to the use of a reduced number of samples, which favors false diagnoses because of nutritional excess^25^.

The efficiency of ACD reached correct diagnoses in more than 90% and 50% of cases for N and K, respectively (Table 6). The high efficiency of the diagnosis of nutritional deficiencies reinforces the crop's high response to fertilization^17,20^, especially for N because most soils are deficient in N. This deficiency could favor an increase in crop productivity. However, the low number of correct diagnoses of K deficiency is worrying, since in areas of low fertility due to this macronutrient, K could decrease the frequency of fertilization of an element, which, according to Zhang et al.^26^, is the most demanded by the crop.

The estimate of the ECS, indicated low precision for N (25%) and high precision for K, reaching 59% (Table 6). This is important because the increase in these indexes in diagnostics eliminates the application of unnecessary fertilizers. If such an application is done, there would be no increase in productivity and practical implications for saving non-renewable fertilizers, improving crop sustainability and probably the economic return of agricultural activity. As for the ACT, the precision was high for N (70%) and low for K (54%). Therefore, a correct diagnosis of nutritional toxicity could also avoid the recommendation of fertilizers. According to Wadt and Lemos^17^, that could aggravate toxicity and consequently lead to a decreased productivity.

When evaluating the GA, we showed that our results partially agreed with those reported by Teixeira et al.^19^ in Brazil because, for N, our result (51%) was closer those the authors reported (63%), unlike for K (33%), whose results were far from those reported by the authors (69%). We emphasize that Teixeira et al.^19^ used the method proposed by Beverly and Hallmark^16^, which considers only three measurements of accuracy, unlike the one we used in this work, which considered seven measurements of accuracy.

A low accuracy of DRIS norms has also been found in other species, although the number of crops is very small, such as sugarcane (*Saccharum officinarum* L.)^20,25,27^ and mango (*Mangifera indica* L.)^28^. Therefore, this issue has to be well discussed in order to take actions to reverse the low precision of diagnoses generated, thus aiming to induce direct benefits in the valuation of the use of leaf analysis in technique-intense crops, favoring sustainability by optimizing the use of most non-renewable fertilizers. Therefore, it is necessary to improve the DRIS method to generate correct diagnoses in any area with a history of different levels of fertility. Our study confirms the need to improve the DRIS diagnostic methods before being used for recommending fertilizers^8^.

In this sense, there are different mechanisms for modifying DRIS norms that could be analyzed in future research. Adjustments could be in the sensitivity coefficients to reduce diagnostic risks due to false deficiency or false insufficiency^28^ or modeling DRIS functions^20^, and using multivariate relationships^28^ and different criteria in the definition of the reference population. Until recently, it was not possible to verify whether changes in the calculation of the DRIS method proposed by different authors would be effective in determining whether there has been any improvement in the quality of the method. There are many studies that have carried out analyses comparing the interpretations of different methods of calculating DRIS norms by comparing them with a diagnosis generated by the critical level. If there were a greater number of concordant diagnoses, the best standard would be chosen^19,24,28^ without testing the plant's response and, therefore, it could not be possible to verify whether the chosen sample could be efficient in diagnoses. However, our work contributes in that it indicates that the measurement of accuracy adopted here allows verifying accuracy using the plant itself as a response and indicating the ability of a specific DRIS standard to generate correct nutritional diagnoses.

Our research proposes the adoption of accuracy measurements to evaluate nutritional diagnoses using DRIS for banana, indicating the need for adjustments to reduce false diagnoses to an acceptable minimum, consequently affecting the optimized use of fertilizers and increasing the sustainability of crops.

## Conclusion

Our study shows that the accuracy method used here is useful for evaluating diagnoses using DRIS norms for banana produced in Ecuador, which depends on the nutrient that is variable with the crop nutritional status. The DRIS norms for diagnosis of N and K result in an acceptable accuracy to identify only deficiencies and toxicities, respectively, indicating the need for adjustments in these standards for later use in the field.

## Methods

### Experimental conditions and plant growth

This study was developed from data collected in 233 commercial banana crops in the province of El Oro, Ecuador, between 2018 and 2020. All selected crops used the cultivars "Vallery" and "Williams" (triploid AAA group). This research was not conducted with endangered species and was carried out in accordance with the Declaration of the IUCN Policy on Research Involving Endangered Species. An average planting density of 1,500 plants ha^− 1^ was adopted. The climate of the region is AW (tropical savannah), according to the Köppen-Geiger classification. The soils in these areas originate from alluvial formation^29^ and are of the order inceptisol, according to the taxonomic classification of the United States Department of Agriculture (USDA)^30^, which is adopted in Ecuador. The cultural treatments carried out in areas cultivated with banana trees, including fertilization**,** phytosanitary control and irrigation, followed the indications of Robinson and Galán-Saúco^31^.

### Chemical analyses of leaves

An annual leaf sampling was carried out on ten healthy and representative plants between January and April 2018, 2019, and 2020, respectively. Leaf collection was performed considering the removal of the central portion (10 cm) of the third leaf (counted from the apex) at the beginning of flowering and in a succession plant (daughter plant) with a height of 1.5 m^32^. Then, the samples were dried in an oven with forced air circulation set to 65ºC until reaching a constant mass. Then, they were ground with a Wiley mill. Next, a chemical analysis was performed to determine the leaf contents of macronutrients (N, P, K, Mg**,** Ca**,** and S) and micronutrients (Cl, Fe, Mn, Cu, Zn, and B) following the methodology described by Bataglia et al.^33^. The samples were washed with in water, then passed through a solution of deionized water and neutral detergent (0.1%), consecutively the samples were washed with a hydrochloric acid solution. Then, dried in a forced ventilation oven at 60 ± 5 °C, up to constant mass of dry matter (DM). To determine the total N content, wet digestion with sulfuric acid was reduced by the semi-micro-Kjeldahl determination method. The elements P, K, Ca, Mg, S, Cu, Fe, Mn and Zn were extracted by digestion with nitric-perchloric acid and determined by atomic absorption spectrometry. On the other hand, B by dry route and Cl by aqueous digestion and stirring.

The banana productivity (PR) was obtained in each property by collecting fruits of ten plants of each plot. The result was expressed in t ha^-1^, following the indications of Rodriguez and Rodriguez^34^.

### Establishment of DRIS norms

The database containing results of leaf analysis and annual average PR was subdivided into populations of high and low productivity (HP and LP). In this context, to define HP, the production limit (PL) was calculated. It consisted of a value corresponding to the mean plus the standard deviation of PR^20^. After defining this parameter, only farms with a PR greater than PL were considered as HP. A data split was performed between the initial basis for calculation of Initial DRIS norms (IBD) and the basis for calculation of DRIS from experimental data (EBD) (Table 1).

The DRIS norms (N-DRIS) were calculated by transforming all 233 observations of leaf contents into % to homogenize the comparison criteria between them. This was done by first sorting the plots according to yield in decreasing order and then the high yield population comprised plots having yield higher than the mean + 0.5 standard deviation, calculated from the entire dataset. Subsequently, logarithmic transformations were applied to the data and the direct and inverse bivariate relationships between all nutrients were determined according to Beverly^35^:

$${\text{Lt }} = {\text{ log }}\left( {{\text{A}}/{\text{B}}} \right)$$

where: Lt = logarithmic transformation of bivariate relationships, log (A/B) = logarithm in which nutrients A and B have a direct relationship.

To determine the functions of the proportion of DRIS nutrients, the physiological diagnosis method and the simplified formula of Beaufils^7^ ${\text{F}}_{{\text{DRIS A}}} = \, \left[ {\sum {\text{f }}\left( {{\text{A }}/{\text{ B}}} \right) \, - \sum {\text{f }}\left( {{\text{B }}/{\text{ A}}} \right)} \right] \, / \, \left( {{\text{n }} + {\text{ m}}} \right)$.

Where: FDRIS A = DRIS function of any nutrient (A); (A / B) = value of the DRIS functions on which nutrients A and B have a direct relationship; (B / A) = value of the DRIS functions in which nutrients B and A have an inverse relationship; n = number of functions in which the nutrient appears in its direct form; m = number of functions in which the nutrient appears inversely.

Subsequently, the Mean Nutritional Balance Index (NBIm) was calculated using the expression:

$${\text{NBIm }} = \, \left| {{\text{I}} - {\text{DRIS A}}} \right| + \left| {{\text{I}} - {\text{DRIS B}}} \right| + \left| {{\text{I}} - {\text{DRIS C}}} \right| + \ldots + \, \left| {{\text{ I}} - {\text{DRIS N}}} \right|/{\text{n}}$$

where: I-DRIS A = DRIS index of any nutrient (A); n = number of DRIS indexes of nutrients included in the analysis.

The interpretation of the PRF was performed using the I-DRIS in three interpretations, equilibrium (Eq) when:

$\left| {{\text{I}} - {\text{DRIS}}} \right| \le {\text{NBIm}}$ , deficiency (D), when: NBIm < │ I-DRIS │ = negative and excess (Ex), when NBIm < │ I-DRIS │ = positive^18^.

The means of high-production subpopulation indexes were considered to establish the nutrient limitation order. Nutritional deficiencies and excesses were obtained by negative and positive indexes, respectively, and the highest levels represented the most limiting nutrients^21^.

### Experimental validation of established DRIS norms

The DRIS norms, were validated experimentally along a banana productive cycle, cultivar “Williams,” of the subgroup Cavendish (*Musa* AAA), from January 2019 to March 2020, in the experimental station Santa Inés, belonging to the Technical University of Machala (El Oro Province), Ecuador (3°17ʹ22'' S, 79°54′43'' W). The climate of the experimental area is tropical savannah (AW), according to the Köppen-Geiger classification.

The experiment considered randomized blocks with four replications in a 4 × 4 factorial design, being four doses of N (0, 200, 400, and 600 kg ha^− 1^) as ammonium nitrate (34% N) and four doses of K_2_O (0, 375, 750, and 1125 kg ha^− 1^) as potassium chloride (60% K_2_O). These nutrient doses were established following an indication around the recommended dose for banana^36^. In addition, the crop received 50 kg ha^− 1^ of P_2_O_5_ as triple superphosphate (46% P_2_O_5_) and 64 kg ha^− 1^ of CaO and 60 kg ha^− 1^ of SO_4_ as calcium sulphate (23% CaO and 18% S). The experimental unit consisted of two rows of nine plants each spaced 2.2 m between rows and 1.7 m between plants, considering only the five central plants in the plot for nutritional and productivity assessment. The total area of the experimental plot, including all treatments and repetitions, under the aforementioned factorial scheme was 0.9 ha.

In the experiment, leaf samples were collected as indicated by Martin-Prevel^32^. Subsequently, the chemical analysis was carried out according to Bataglia et al.^33^ and the contents of macronutrients (N, P, K, Mg, Ca, and S) and micronutrients (Cl, Fe, Mn, Cu, Zn, and B) were determined. The PR was calculated by the multiplication of the mass of bunches harvested from plants by the population density expressed in t ha^− 1^. The harvest was carried out manually approximately 36 weeks after sowing. After obtaining the leaf contents and the PR, another database for DRIS calculation of the experiment was created and, together with the DRIS norms previously determined, the I-DRIS and NBIm was calculated according to Beaufils^7^, PRF according to Wadt^18^ and the nutrient limitation order according to Abebe et al.^21^.

The leaf nutritional diagnoses of N and K were evaluated by a percentage increase in PR (I_PR%), based on the plant's response to fertilization with N and K_2_O in relation to a control condition considering increases or decreases equal to or above 10% in PR^20^. The value was considered in nutritional equilibrium (Eq) when I_PR% ≤ 10%, nutritional deficiency (D) when I% _PR > 10%, and nutritional excess (Ex) when I% _PR < − 10%^16^.

### Accuracy of nutritional diagnoses

To model DRIS formulas, the variable F was introduced to interpret the indexes calculated by DRIS, adopting the value of F = 1.0, as described by Silva et al.^20^. Then, the accuracy of the nutritional status diagnosis for the nutrients N and K was determined. It was defined by two classes of interpretation, deficient (D) and sufficiency (S), using the DRIS norms created in this study.

The accuracy assessment consisted of verifying whether the nutritional diagnosis, obtained by the DRIS procedure, corresponded to the crop response depending on the PR variation, when N and K_2_O were applied in the experiment, always comparing the values with those of a control situation (without applying the two elements to the soil). In this context, the pairs of plots from a same experimental block were compared and received the same treatments, except for variations in N and K fertilizers**.** The diagnoses of the control situation were classified into D and S, as previously described, following the criterion for interpreting DRIS indexes using the PRF method^18^.

For the assessment of nutritional diagnoses, predictive diagnostic analyses for fertilization were used on a scale of 0 to 1, and contrasted with the seven accuracy measurements proposed by Wadt and Lemos^17^, considering three possible nutritional states: deficiency, sufficiency and toxicity.

### Handling of plants

The authors confirm that the handling of the plants is accordance with the Declaration of IUCN Policy on Research Involving Endangered Species and the Convention on Trade in Endangered Species of Wild Fauna and Flora.

## Data Availability

All data generated or analysed during this study are included in this manuscript.
